# Deciphering chemokine properties by a hybrid agent-based model of *Aspergillus fumigatus* infection in human alveoli

**DOI:** 10.3389/fmicb.2015.00503

**Published:** 2015-05-28

**Authors:** Johannes Pollmächer, Marc Thilo Figge

**Affiliations:** ^1^Applied Systems Biology, Leibniz-Institute for Natural Product Research and Infection Biology – Hans Knöll InstituteJena, Germany; ^2^Faculty of Biology and Pharmacy, Friedrich Schiller University JenaJena, Germany

**Keywords:** *Aspergillus fumigatus*, fungal infection, agent-based modeling, reaction-diffusion equation, chemotaxis, human alveolus, alveolar macrophage, alveolar epithelial cell

## Abstract

The ubiquitous airborne fungal pathogen *Aspergillus fumigatus* is inhaled by humans every day. In the lung, it is able to quickly adapt to the humid environment and, if not removed within a time frame of 4–8 h, the pathogen may cause damage by germination and invasive growth. Applying a to-scale agent-based model of human alveoli to simulate early *A. fumigatus* infection under physiological conditions, we recently demonstrated that alveolar macrophages require chemotactic cues to accomplish the task of pathogen detection within the aforementioned time frame. The objective of this study is to specify our general prediction on the as yet unidentified chemokine by a quantitative analysis of its expected properties, such as the diffusion coefficient and the rates of secretion and degradation. To this end, the rule-based implementation of chemokine diffusion in the initial agent-based model is revised by numerically solving the spatio-temporal reaction-diffusion equation in the complex structure of the alveolus. In this hybrid agent-based model, alveolar macrophages are represented as migrating agents that are coupled to the interactive layer of diffusing molecule concentrations by the kinetics of chemokine receptor binding, internalization and re-expression. Performing simulations for more than a million virtual infection scenarios, we find that the ratio of secretion rate to the diffusion coefficient is the main indicator for the success of pathogen detection. Moreover, a subdivision of the parameter space into regimes of successful and unsuccessful parameter combination by this ratio is specific for values of the migration speed and the directional persistence time of alveolar macrophages, but depends only weakly on chemokine degradation rates.

## 1. Introduction

*Aspergillus fumigatus* is the most dangerous airborne fungal pathogen in humans leading to high mortality rates (Heinekamp et al., 2014). Immunocompetent individuals are able to prevail over inhaled conidia of the fungus in an everyday challenge. In contrast, patients with an altered immune system, e.g., as a consequence of organ transplantation or an underlying disease like HIV, are at high risk to die from invasive aspergillosis (Horn et al., 2012), where the lung is the site of infection in 70 % of the cases (Lin et al., 2001). *A. fumigatus* is able to adapt within hours to the humid and nutrient rich milieu of the lung (Hohl, 2008; Hasenberg et al., 2011), by this setting a tight time scale for phagocytes to find, detect and remove the pathogenic fungus before the onset of germination and hyphal invasion of alveolar epithelium.

Alveolar macrophages (AM) reside on the inner surface of lung alveoli and are the first professional motile phagocytes that get in contact with inhaled conidia of *A. fumigatus* (Hasenberg et al., 2013). AM are capable of clearing the lower respiratory tract from all kinds of inhaled particles and microbes in order to maintain a pathogen-free alveolar surface and to ensure optimal exchange of oxygen and carbon-dioxide (Fels and Cohn, 1986). The migration of AM takes place within the alveolar lining layer, which is a viscous fluid—referred to as surfactant—that coats the alveolar surface with an average thickness of about 200 nm (Bastacky and Lee, 1995). Apart from the stabilizing effect of the surfactant avoiding alveolus collapse, it also provides the environment for diffusive transport of molecules, such as lipids and immunoregulatory proteins SP-A and SP-D, that are continuously produced, secreted and recycled by alveolar epithelial cells (AEC) (Herzog et al., 2008).

For over one decade computational approaches have proven to successfully complement wet-lab studies in the frame of systems biology (Kitano, 2002; Horn et al., 2012). Computer modeling and simulation are nowadays important tools to verify hypotheses in advance of cost- and time intensive experimental investigations to narrow down the range of possible wet-lab experiments to the most promising ones. Furthermore, predictions may be derived from virtual models, which subsequently can be tested in experiment. The present study aims at predicting AM chemokine properties from an existing agent-based virtual infection model of human alveoli under physiological conditions (Pollmächer and Figge, 2014). Due to the peculiar physiology of the human lung, investigations *in vivo*, including live-cell imaging, are hard to realize. Thus, quantitative measures like AM motility, chemokine secretion rates of AEC or the diffusion coefficient of molecules within the surfactant are not directly accessible. AEC type II cell lines have been studied intensively in the past, but as they do account for only five percent of the alveolar surface, experimental investigations of type I AEC would be highly appreciated. However, isolation and cultivation of type I AEC with current methods are demanding tasks due to their thin and delicate morphology. The present computational modeling approach enables us studying *A. fumigatus* infection in alveoli for varying parameter sets of AM motility and of chemokine properties in order to reveal the relative importance of each of the parameters and their potential regimes in healthy individuals.

Recently, we established an agent-based model (ABM) of *A. fumigatus* infection in the human alveolus to study the early immune response under physiological conditions (Pollmächer and Figge, 2014). In this three-dimensional to-scale model, we represented the human alveolus by a three-quarter spherical structure consisting of type I and type II AEC as well as pores of Kohn. Our computations of the first-passage-time, i.e., the time it takes until the conidium is detected by an AM for the first time, clearly showed that pathogen detection by AM resembles the problem of finding the needle in the haystack within a time limit that is set by the germination time for *A. fumigatus* conidia of about 6 h. Statistical analyses based on hundreds of thousands of computer simulations revealed that for AM to successfully accomplish finding the conidium within 6 h time, chemotactic cues are required that guide AM to the AEC associated with a conidium. Chemotaxis was implemented in the ABM based on a probabilistic rule, i.e., AM were directed toward the AEC associated with the fungus with a probability that was defined by the distance-dependent strength of the chemokine gradient (Pollmächer and Figge, 2014). The gradient of the chemokine concentration in the alveolus was approximated by the analytical steady state solution of the two-dimensional diffusion equation for a point source on a planar surface. We demonstrated that this level of detail was sufficient to arrive at the conclusion that chemotactic cues are required for directing AM migration in the alveolus to the site of the pathogen. However, the specific chemokine remains as of yet unknown, including its characteristic parameters such as the secretion rate, diffusion coefficient and rate of degradation. In order to arrive at quantitative predictions of characteristic parameters that may narrow down the regime of candidate chemokines, the ABM has to be revised to describe the spatio-temporal dynamics of chemokine diffusion in the alveolus and the receptor binding on AM at a higher level of detail.

**Figure 1:**
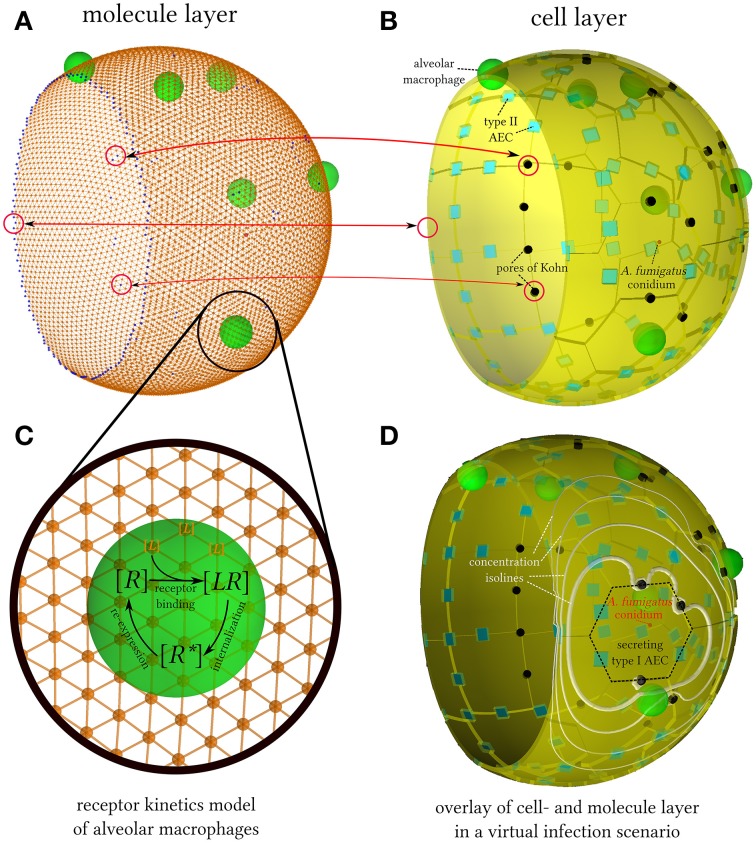
**Schematic overview and structural relations between different components of the hybrid agent-based model**. **(A)** Close-to-equidistant discretization of the three-quarter alveolus with 10,000 grid points. Grid points with label alveolar surface point (*orange spheres*) are connected with their neighboring grid points (*orange lines*) and those with label boundary point (*blue spheres*) correspond to either pores of Kohn or the alveolar entrance ring. **(B)** To-scale reconstruction of the human three-quarter alveolus from Pollmächer and Figge (2014) including alveolar epithelial cells (AEC) of type I (*yellow*) and type II (*blue*) as well as the pores of Kohn (*black*). **(C)** Receptor kinetics model that drives the chemotaxis of alveolar macrophages (AM). Free chemokine receptors [*R*] bind to chemokine ligands [*L*] located at grid points associated with the AM. Bound receptors [*LR*] are processed into internalized receptors [R^*^] and are re-expressed subsequently. **(D)** Snapshot of a virtual infection scenario where AM (*green*) aim to find a conidium of *A. fumigatus* (*red*). The information contained in the molecule layer is integrated using chemokine concentration isolines (*white*), which are plotted proportional to their respective values with different sizes.

Mathematical models of chemotaxis typically set focus on one of the three key aspects that are associated with the directed migration of cells: gradient-sensing, polarization and motility. While integrative models combining all three aspects are still rare today (Iglesias and Devreotes, 2008), a chemotaxis model including the processes of gradient-sensing and motility was developed by Guo and Tay (2008). In this approach, a hybrid ABM (hABM) was used to simulate the migration behavior of leucocytes and to compare with experimental results of under-agarose assays. A hABM is a multi-scale model where cells are represented as migrating and interacting agents that are coupled to the interactive layer of diffusing molecule concentrations by the kinetics of chemokine receptor binding, internalization and re-expression (see Figure 1). From a technical point of view, this requires the implementation of a solver for the spatio-temporal reaction-diffusion equation of molecule concentrations in the complex alveolar structure with spherical symmetry and peculiar boundary conditions as imposed by the pores of Kohn and the alveolar entrance ring. This is achieved by generating a Delaunay triangulation of the alveolar surface for close-to-equidistant surface points. The geometric quantities of the corresponding Voronoi tesselation, i.e., the dual graph of the Delaunay triangulation, can then be used to solve the reaction-diffusion equation by a finite difference method on unstructured grids (Sukumar, 2003). We perform a numerical study of the steady state behavior of molecules for typical values of the diffusion coefficient, chemokine secretion rate and the rate of molecular degradation. Furthermore, performing statistical analyses of first-passage-time distributions we narrow down the regime of characteristic parameters required for the time-limited detection of *A. fumigatus* conidia by AM.

## 2. Materials and methods

### 2.1. Hybrid agent-based model

In this study, we revised our agent-based model (ABM) of the human alveolus to explicitly account for the dynamics of molecular diffusion and reactions with cells, which were previously modeled in a simple rule-based fashion using a steady-state approximation (Pollmächer and Figge, 2014). We refer to the revised model as hybrid agent-based model (hABM), because single cells are represented as individual agents that migrate and interact in continuous space, whereas chemokine concentrations are represented as spatio-temporal distributions on a discrete grid. In this multi-scale approach, interactions between cellular agents and the layer of diffusing molecular concentrations are realized via modeling the kinetics of chemokine receptor binding, internalization and re-expression on alveolar macrophages (AM) as shown in Figure 1. The present agent-based simulation algorithm has linear time complexity in the number of agents and in the number of timesteps. Thus, treating single molecules as single virtual agents would render the simulations computationally intractable. Scalability in terms of constituent quantities is one of the strengths of partial differential equations (Horn et al., 2012) as the time complexity of our numerical method is linear in the number of grid points, molecule species and timesteps. In summary, treating cells at the microscopic level of discrete agents and molecules at the macroscopic level of continuous distributions ensures keeping the balance between computational tractability and detailed modeling across interwoven time- and length-scales (Guo et al., 2008). The source code of the hABM is available from the authors upon request.

### 2.2. Numerical solution of the reaction-diffusion equation in the alveolus

#### 2.2.1. Reaction-diffusion equation

The spatio-temporal distribution of chemokines on the inner surface of the alveolus is described by the following reaction-diffusion equation:

(1) $$\frac{\partial c(\vec{r},t)}{\partial t}=D\Delta c(\vec{r},t)-\lambda c(\vec{r},t)+S(\vec{r},t)-Q(\vec{r},t).$$

Here *c*($\vec{r}$, *t*) denotes the molecular concentration of chemokines at position $\vec{r}$ and time *t* and Δ is the Laplace operator. The chemokine's isotropic diffusion coefficient is given by *D* and its degradation rate is given by λ. The spatio-temporal source of molecular concentration is represented by the term *S*($\vec{r}$,*t*) associated with chemokine producing alveolar epithelial cells (AEC) of type I and type II. The term *Q*($\vec{r}$,*t*) represents the uptake of chemokines by AM and is explained in detail below. Numerical integration of the reaction-diffusion Equation (1) within the surfactant on the inner alveolar surface requires a discretization of the thin fluidic lining layer by a grid with close-to-equidistant grid points.

#### 2.2.2. Discretization of the surfactant

Generating a grid with an arbitrary number of close-to-equidistant grid points on the surface of a spherical geometry is related to the Thomson problem (Thomson, 1904). This problem was raised more than a century ago in the context of finding the minimal electrostatic potential energy configuration for *n* equally charged particles that repel each other by Coulomb forces on the surface of a unit sphere. An equidistant distribution of points is beneficial for the numerical solution of the reaction-diffusion equation with regard to computing time and numerical stability. We take advantage of a crowd-based numerical approximation platform that determines the global minima using a variety of different optimization algorithms (MacWilliam and Cecka, 2013). Next, in order to obtain the neighborhood relationship between the grid points, we use the close-to regular distribution of points as inputs and compute the convex hull, where each of its edges corresponds to a pair of neighboring grid points. Note that the triangulation of discrete points on a sphere surface using the convex hull is equivalent to the Delaunay triangulation of these points in three dimensions (Brown, 1979). Finally, the dual graph of the Delaunay triangulation, i.e., the Voronoi tesselation (De Berg et al., 2008), was computed in order to obtain the surface-area associated with each grid point, i.e., the area of the corresponding Voronoi cell. As will be shown below, this measure together with the length of the Voronoi edge between neighboring Voronoi cells are required for solving the reaction-diffusion Equation (1) numerically.

It should be noted that, since the human alveolus does not correspond to a full sphere, not each grid point belongs to the alveolar surface. In fact, each point of the grid can be labeled as one of the three categories: (i) alveolar surface point, (ii) boundary point, (iii) outside point. A point is considered to be an alveolar surface point if it is part of the alveolar three-quarter sphere and does not cover a pore of Kohn. All other points are outside points, except for boundary points which have at least one neighboring point being an alveolar surface point (see Supplementary Figure S1A and Video S1). We use absorbing boundary conditions in each simulation scenario, i.e., the concentration at each boundary point is kept fixed at zero for all times. The representation of the surfactant with an average thickness of only 200 nm (Bastacky and Lee, 1995) is based on 10^4^ close-to-equidistant grid points of the spherical surface at an average distance of 4.45 ± 0.16 μm (see Video S1). This allows resolving AEC of type I and type II that are, respectively, 60 μm and 9.3 μm in diameter, as well as the pores of Kohn that are 6 μm in diameter as estimated from literature data in Pollmächer and Figge (2014).

#### 2.2.3. Numerical integration of the reaction-diffusion equation

The reaction-diffusion Equation (1) is numerically integrated in time using a finite difference method for unstructured grids as described by Sukumar (2003). Here, Voronoi cells are the placeholders of the chemokine concentrations, where each Voronoi cell may contain several molecular species. The *k*th Voronoi cell is associated with grid point $\vec{r}$_*k*_ of the Delaunay triangulation and has area *A*_*k*_ and a finite set of neighbors 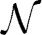
(*k*). The relation with neighboring Voronoi cells ℓ ∈ 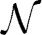
(*k*) is defined by the length of the Voronoi edge *h*_*k*ℓ_ and the Euclidean distance between the two Voronoi cells *d*_*k*ℓ_, as depicted in Supplementary Figure S1B. The numerical integration is then performed in a straightforward fashion over each Voronoi cell *k* that is associated with a grid point of the category alveolar surface point: 
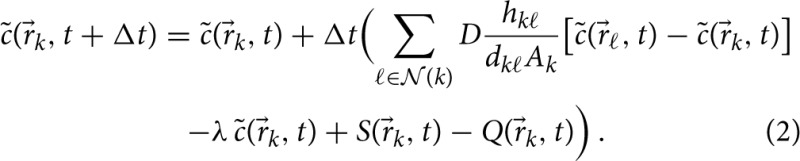


Here and in what follows the discretized concentration values are indicated by the symbol $\tilde{c}$. In our model, both AEC of type I and type II may secrete chemokines, which is appropriately captured by a non-vanishing source term *S*($\vec{r}$_*k*_, *t*) at all grid points of the AEC associated with the conidium.

#### 2.2.4. Validation of the numerical solution

In order to validate the implementation of the close-to-equidistant grid for the spherical system and the algorithm for the numerical solution of the reaction-diffusion Equation (1), we performed simulations of scenarios for which the analytical solutions are known. These scenarios were based on the analytical solution of the isotropic diffusion equation in terms of spherical harmonics (Sbalzarini et al., 2006). For a sphere with radius *r* and molecular diffusion coefficient *D* on its surface an analytical solution of the reaction-diffusion Equation (1) for vanishing molecule degradation and absent source- and reaction-term is given by

(3) $$c(\vec{r}=(r,\vartheta ,\varphi ),t)=\sqrt{\frac{3}{4\pi }}\cos(\vartheta )\exp\left(-\frac{2D}{r^{2}}t\right)\;.$$

Here, surface positions $\vec{r}$ are represented using spherical coordinates with polar angle ϑ and azimuthal angle φ. Simulations were started from the initial condition $c(\vec{r},t=0)=\sqrt{3/(4\pi )}\cos(\vartheta )$. The accuracy of the numerical solution was evaluated by comparing with the analytical solution on the spherical surface using biquadratic interpolation at 2× 10^4^ pre-defined close-to-equidistant points.

### 2.3. Chemotaxis model of alveolar macrophages

The previously established agent-based model of the human alveolus (Pollmächer and Figge, 2014) is extended by modeling the interactions between molecule concentrations and chemokine receptors of AM, including the internalization of bound receptors and their subsequent re-expression on the AM surface. This enables AM to sense chemokine gradients that ultimately drive the migratory response of the phagocytes. Here, we essentially follow the receptor kinetics model as previously presented in Guo and Tay (2008) and Guo et al. (2008), apart from modifications required in the present context of modeling the dynamics of infection in the curved environment of a human three-quarter alveolus.

Since the average distance between neighboring grid points is four to five times smaller than the AM diameter of *r*_AM_ = 10.6 μ*m* (Krombach and Münzing, 1997), each AM is an agent associated with on average 20 grid points on the interactive molecule layer. In the reaction-diffusion Equation (1), the interaction between chemokines and AM receptors is represented by the term 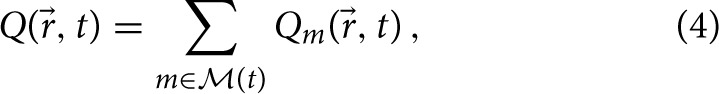 where 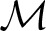
(*t*) is the set of AM present in the alveolus at time *t*. *Q*_*m*_($\vec{r}$, *t*) denotes the reaction term of the *m*th AM with the chemokines in the surfactant, which is defined at each grid point *q* as follows:

(5) $$Q_{m}({\vec{r}}_{q},t)=\{\begin{array}{l} \frac{k_{b}}{A_{\text{AM}}}\tilde{c}({\vec{r}}_{q},t){\left[R\right]}_{m}(t)\text{ , if}q\in {\text{cov}}_{m}(t)\\ 0\text{ otherwise,} \end{array}$$

where cov_*m*_ (*t*) represents the set of covered grid points by the *m*th AM (see Supplementary Figure S2), [*R*]_*m*_(*t*) is the current number of free receptors on the AM and *k*_*b*_ is the binding rate between AM receptors and the chemotactic cytokines in the surfactant. The interaction surface for the reaction between the receptors of the AM cell wall and the chemokines in the surfactant is denoted by *A*_AM_ = π *r*^2^_AM_. Beside the number of free receptors, each AM *m* is an agent keeping track of its current number of bound ([*LR*]_*m*_) and internalized receptors ([R^*^]_*m*_). The kinetics of ligand-binding, receptor internalization and re-expression is described by a system of ordinary differential equations:

(6) $$\frac{\text{d}{\left[R\right]}_{m}(t)}{\text{d}t}=k_{r}{\left[R^{*}\right]}_{m}(t)-A_{\text{AM}}\sum_{q}Q_{m}({\vec{r}}_{q},t)\;,$$

(7) $$\frac{\text{d}{\left[RL\right]}_{m}(t)}{\text{d}t}=A_{\text{AM}}\sum_{q}Q_{m}({\vec{r}}_{q},t)-k_{i}{\left[LR\right]}_{m}(t)\;,$$

(8) $$\frac{\text{d}{\left[R^{*}\right]}_{m}(t)}{\text{d}t}\;=\;k_{i}{\left[LR\right]}_{m}(t)-k_{r}{\left[R^{*}\right]}_{m}(t)\;.$$

Here, *k*_*i*_ is the internalization rate of bound receptors and *k*_*r*_ is the recycling rate associated with the re-expression of internalized receptors. All model parameters together with their experimentally relevant regimes of values are listed in Table 1. The parameters related to the receptor-kinetics model of AM, *k*_*b*_, *k*_*i*_ and *k*_*r*_, are fixed to the geometric means of their corresponding experimental range.

**Table 1:** **Parameters used for the chemotaxis model of alveolar macrophages**.

**Symbol**	**Description**	**Unit**	**Value**	**Experimental range**	**References**
*D*	Chemokine diffusion coefficient (in water)	μm^2^ × min^−1^	Varied	6×10^2^ – 3.5×10^4^	(Francis and Palsson, 1997; Randolph et al., 2005)
*s*_AEC_	Chemokine secretion rate of AEC	min^−1^	Varied	Unknown	
λ	Chemokine degradation rate	min^−1^	Varied	3× 10^−3^ – 4.2× 10^−2^	(Beyer and Meyer-Hermann, 2008)
*k*_*b*_	Ligand-receptor binding rate	μm^2^ × min^−1^	1× 10^−2^	7× 10^−4^ – 0.3	(Sklar, 1984; Pelletier, 2000; Guo et al., 2008)
*k*_*i*_	Receptor internalisation rate	min^−1^	7× 10^−2^	3× 10^−3^ – 1.8	(Beyer and Meyer-Hermann, 2008; Guo et al., 2008)
*k*_*r*_	Receptor recycling rate	min^−1^	5× 10^−2^	6× 10^−3^ – 0.5	(Beyer and Meyer-Hermann, 2008; Guo et al., 2008)
*R*_0_	Initial number of chemokine receptors		5×10^4^	2×10^4^ – 2×10^5^	(Beyer and Meyer-Hermann, 2008; Guo et al., 2008)
σ_AM_	Sensitivity to bound-receptor differences		1.2×10^−3^	1.2× 10^−3^	(Devreotes and Zigmond, 1988; Farrell et al., 1990)

In our model, the kinetics of bound receptor differences along the current chemokine gradient is coupled to the directional persistence time of migrating AM (Farrell et al., 1990). Thus, as shown in Supplementary Figure S2, we consider that the *m*th AM weights the direction of the average chemokine concentration gradient $\vec{g}$_*m*_(*t*) in each timestep by the difference in newly bound receptors at the front and rear of the cell along the gradient.

The difference in the chemokine concentration across the interaction surface of the *m*th AM between its front and rear, Δ*c*_*m*, diff_, is computed using the distance between the respective barycenters of the front und rear of this AM and its corresponding concentration gradient:

(9) $$\Delta c_{m,\text{diff}}(t)=\frac{8r_{\text{AM}}}{3\pi }||{\vec{g}}_{m}(t)||\;,$$

where the chemokine concentration gradient $\vec{g}$_*m*_(*t*) over the *m*th AM is obtained from averaging over the local gradients of all grid points covered by the *m*th AM (cov_*m*_(*t*)). Then, the difference in newly bound receptors between the front and rear of the AM along the current gradient per timestep Δ*t* is

(10) $$\Delta {\left[LR\right]}_{m,\text{diff}}(t)=k_{b}\Delta c_{m,\text{diff}}(t)\frac{{\left[R\right]}_{m}(t)}{2}\Delta t\;.$$

The most favorable direction of migrating AM is determined by computing the sum of weighted gradients over one period of directional persistence:

(11) $${\vec{g}}_{m,\text{cum}}(t_{\text{begin}}^{*},t_{\text{end}}^{*})=\sum_{t=t_{\text{begin}}^{*}}^{t_{\text{end}}^{*}}\Delta {\left[LR\right]}_{m,\text{diff}}(t)\frac{{\vec{g}}_{m}(t)}{\left||{\vec{g}}_{m}(t)|\right|}\;,$$

where t^*^_begin_ and t^*^_end_ denote the start and the end time for the period of directional persistence.

Finally, after each period of directional persistence, the respective AM migrates in the direction of the weighted cumulative gradient $\vec{g}$_*m*, cum_(t^*^_begin_, t^*^_end_) with probability

(12) $$p_{\text{directed}}=\min(||{\vec{g}}_{m,\text{cum}}(t_{\text{begin}}^{*},t_{\text{end}}^{*})||\sigma_{\text{AM}},1)\;.$$

This probability is proportional to the bound receptor differences along the cumulative gradient (Devreotes and Zigmond, 1988) and the constant of proportionality is the AM sensitivity σ_AM_ that was determined by Tranquillo et al. (1988) (see Table 1).

### 2.4. System setup for simulation studies

#### 2.4.1. Steady state analysis

Initially, all grid points were set to zero molecular concentration and one permanently and homogenously secreting AEC of type I at the bottom of an otherwise empty three-quarter alveolus was placed. Keeping track of the time-dependent relative concentration change,

(13) $$\Delta {\tilde{c}}_{\text{rel}}({\vec{r}}_{k},t)\equiv \frac{\tilde{c}({\vec{r}}_{k},t+\Delta t)-\tilde{c}({\vec{r}}_{k},t)}{\tilde{c}({\vec{r}}_{k},t)},$$

at grid points *k*, the steady state of the molecular distribution was considered to be reached when the maximum value of Δ$\tilde{c}$_rel_ over all grid points fell below a threshold value of one permille. Measurements were repeated 50 times per parameter configuration and the results were averaged, keeping the number of randomly positioned pores of Kohn in the alveolus constant.

#### 2.4.2. Virtual infection scenario

For studying *A. fumigatus* infection in a three-quarter alveolus with constant radius, the virtual infection scenario from Pollmächer and Figge (2014) was followed. At *t* = 0 a binomially distributed number of AM and the conidium were placed randomly over the surface of the three-quarter alveolus and all grid points were set to zero molecular concentration. AM migrated according to a biased persistent random walk with constant speed *v* and constant directional persistence time *t*_p_ and were able to leave or enter the alveolus at either a pore of Kohn or the alveolar entrance ring. The position of the conidium was fixed over the whole simulation and migration of AM followed the chemotaxis model that was here previously introduced. In each virtual infection scenario the AEC of type I or II that was associated with the randomly positioned conidium released the chemoattractant permanently and homogenously with a constant secretion rate *s*_AEC_. The simulation ended at the first physical contact between an arbitrary AM and the conidium. The diffusion coefficient *D* of the chemokine was varied over a wide range in order to account for the viscosity of the surfactant that is expected to be higher than that of water and to which experimental ranges are typically referring. In Table 1, the parameter regimes of the chemotaxis model are summarized and the values that were varied in the simulations are indicated.

#### 2.4.3. Virtual infection scenario including gradient-based recruitment of alveolar macrophages

In Pollmächer and Figge (2014), AM insertion into the three-quarter alveolus followed a uniform distribution over the length of the boundary line elements. Numerical values of chemokine concentrations allow for recruitment of AM from neighboring alveoli based on the strength of the gradient. Realization of gradient-based recruitment was implemented in the following way. First, on AM entrance into the alveolus the maximum gradient was computed, max{||$\vec{g}$_b_(*t*)||}, over the finite set of edges of the triangulated grid that cross the boundary. The pairs of vertices corresponding to these edges each held one vertex labeled as boundary point and the other one labeled as alveolar surface point. Secondly, a uniformly distributed random boundary point $\vec{r}$_b, random_ was drawn from all possible boundary points and the corresponding probability of AM insertion was calculated as follows:

(14) $$p_{\text{in}}({\vec{r}}_{\text{b},\text{random}},t)=\frac{\left||\vec{g}({\vec{r}}_{\text{b},\text{random}},t)|\right|}{\text{max}\{||{\vec{g}}_{\text{b}}(t)||\}}.$$

This probability was used for stochastic AM insertion at position $\vec{r}$_b, random_ and was realized by a Monte Carlo acceptance-rejection method to sample the gradient-based probability distribution of AM insertion over the boundary points. On rejection of a boundary point a new one was drawn with probability *p*_in_($\vec{r}$_b, random_, *t*) followed by another Monte Carlo decision until a boundary point was accepted. As before, first-passage-time simulations were performed over 10^3^ repetitions for each parameter configuration.

## 3. Results

### 3.1. Hybrid agent-based model reproduces analytical solutions

**Figure 2:**
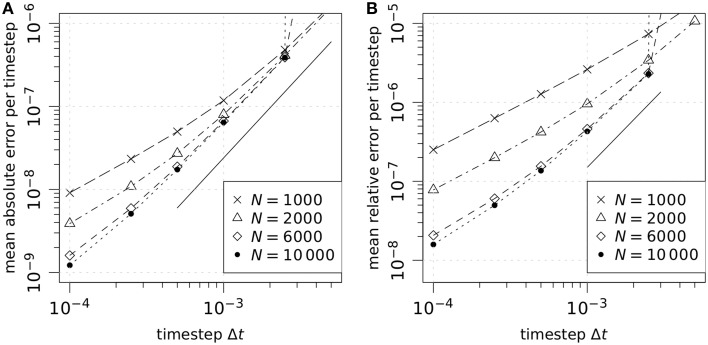
**Numerical error analysis of the PDE solver for Equation (2) on the spherical surface with λ = 0,** ***S*****($\vec{r}$**_***k***_**,** ***t*****) = 0, and** ***Q*****($\vec{r}$**_***k***_**,** ***t*****) = 0 at each grid point** ***k***. Simulations were carried out in an alveolus with a radius *r* = 116.5 μm from time *t* = 0 min to a final time *t* = 1 min with an isotropic diffusion coefficient of *D* = 2000 μm^2^/min. The mean absolute error **(A)** and mean relative error **(B)** per timestep of our PDE solver for different numbers of grid points *N* and timesteps Δ*t* are compared to the theoretically expected quadratic scaling (*solid line*).

We evaluated and validated the numerical solution of our PDE solver by comparison with an analytical solution over the surface of a full sphere (see Section 2.2.4 for details). The mean of the absolute and relative errors per timestep were computed for both varying timesteps and varying numbers of grid points in order to demonstrate the accuracy of the numerical method (see Figure 2). The method shows first-order accuracy in the timestep as the absolute and relative mean errors per timestep scale quadratically. Furthermore, it is observed that numerical instability occurs for too large values of Δ*t*, as is expected for an explicit forward-Euler approach. To guarantee numerical stability in our simulations, we determined the limits of numerical stability for different diffusion coefficients *D* over the set of grid points 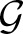 using the condition 
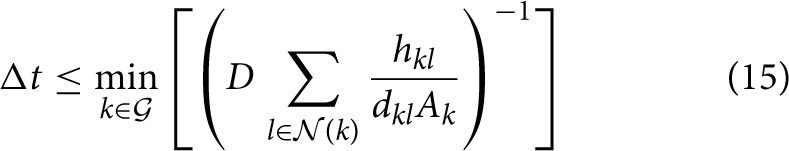
 and adjusted the global simulation timestep Δ*t* one order of magnitude lower than the respective limits.

### 3.2. Steady state of alveolar chemokine distribution reached within hours

**Figure 3:**
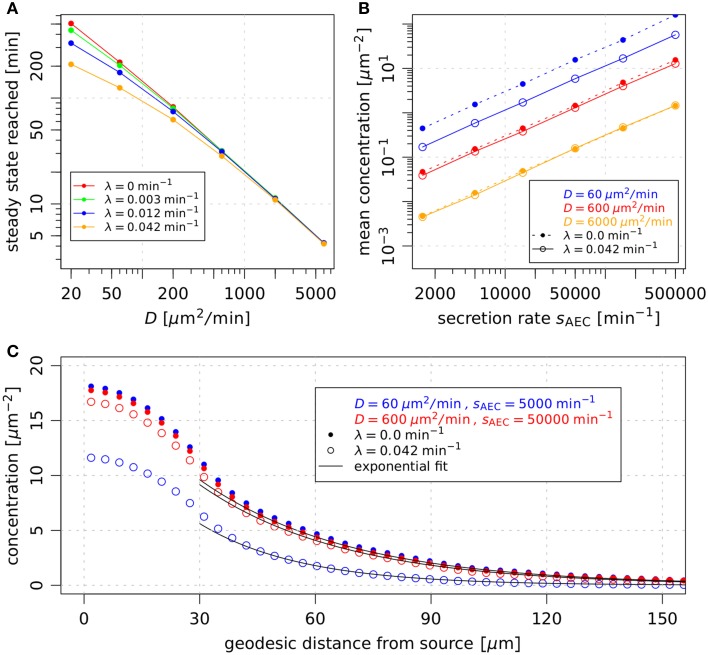
**Steady state analysis of the concentration profile in the alveolus for varied diffusion coefficients** ***D*****, secretion rates** ***s*****_AEC_ and degradation rate λ**. One permanently and homogenously secreting source with radius *r*_AEC_ = 30 μm was placed in the bottom of the three-quarter alveolus and tthe relative concentration changes Δ$\tilde{c}$_rel_ (see Equation 13) were tracked over time at each grid point *k*. The steady state of the molecular distribution was considered to be reached when the maximum value of Δ$\tilde{c}$_rel_ over all grid points fell below a threshold value of 0.001. **(A)** Comparison of the mean values of the time when steady state was reached for different degradation rates and diffusion coefficients averaging over the secretion rates {1.5 × 10^3^, 5 × 10^3^, 1.5 × 10^4^, 5 × 10^4^, 1.5 × 10^5^, 5 × 10^5^} min^−1^. Each mean value has a relative standard deviation less than five percent. **(B)** Average concentration over all grid points labeled as alveolar surface point at steady state. **(C)** Concentration profile at steady state as a function of the geodesic distance from the center of the source. In each simulation concentration values were averaged over points of the three-quarter sphere with equivalent geodesic distance from the center of the source. Here biquadratic interpolation was used to obtain the concentration value at arbitrary points on the alveolar surface. Afterwards the means over simulation runs with identical parameter configuration were computed. We applied exponential fits to each concentration profile using least squares to optimize the parameters *a* and *b* in the function *c*(*x*) = *a* exp(*bx*) over concentration values at geodesic distances above the AEC radius *r*_AEC_ = 30 μm.

We performed a numerical study to characterize the steady state of the alveolar chemokine distribution in terms of the concentration profile and the time required to reach the steady state (see Video S2 for the transition from the onset of AEC secretion into steady state). Simulations were carried out using one permanently and homogenously secreting AEC of type I in the bottom of an empty alveolus (see Section 2.4.1 for details) In Figure 3, we summarize the results of the steady state analysis for varied diffusion coefficients, degradation rates and secretion rates of the chemokine. Interestingly, we found that the time required to reach the steady state depends on the values for the diffusion coefficient *D* and the degradation rate λ but not on the amount of chemokine secretion *s*_AEC_ per time (Figure 3A). In the absence of degradation, the time required to reach the steady state ranges from 4 min for a diffusion coefficient of *D* = 6000 μm^2^/min to 8.5 h for a diffusion coefficient of *D* = 20 μm^2^/min. In the presence of degradation, the times required to reach the steady state were systematically decreasing with increasing degradation rates in a diffusion-dependent fashion (Figure 3A).

In Figure 3B it can be seen that the parameter variation lead to average concentration values that span a range of five orders of magnitude from 10^−2^ μm^−2^ to 10^2^ μm^−2^. The mean concentration was observed to increase linearly with increasing secretion rate *s*_AEC_. We found that different parameter combinations showed similar mean concentration values and almost identical concentration profiles over the geodesic distance from the secreting AEC (see Figure 3C). Irrespective of the secretion rate, diffusion coefficient and degradation rate the profile of concentration over the surface of the alveolus showed an exponential distance-dependence from the secreting AEC for geodesic distances larger than the radius of the secreting AEC.

We generally observed that the impact of chemokine degradation on the time to reach the steady state and on the amount and profile of the chemokine concentration is largest for small diffusion coefficients (see Figures 3A–C). This is a direct consequence of reduced molecule motion, because on average molecules remain in the alveolus for a longer time period before leaving through a pore of Kohn or through the alveolar entrance ring. In particular, the time required to reach the steady state, the average chemokine concentration as well as the level of the concentration profile were lowered for elevated degradation rates. These effects were depending on the diffusion coefficient: While for diffusion coefficents *D* ≥ 2000 μm^2^/min all three observables were reduced by less than 5% relative to the case with absent degradation, for *D* ≤ 60 μm^2^/min this reduction was observed to increase up to 85%. For example, in the extreme case of the small diffusion coefficient *D* = 20 μm^2^/min and at a secretion rate of 1.5× 10^4^ molecules per minute, the average concentration ranges between 2.3 and 14.7 molecules per μm^2^ and the time required to reach the steady state varied in a degradation-dependent fashion between 3.5 and 8.5 h.

### 3.3. Virtual infection model reveals relevant parameter regimes

We performed computer simulations on the early immune response against *A. fumigatus* infection mediated by chemokines that are released from the AEC associated with the conidium. In contrast to our previous study, where chemotaxis was modeled in a simplified fashion by a probabilistic rule (Pollmächer and Figge, 2014), we here implemented a numerical solver for the reaction-diffusion equation extending over the inner surface of the alveolus. Thus, in the present implementation AM performed a biased persistent random walk and the directional bias was derived from local sensing of the current chemokine gradient by AM. The relative impact of directional over random migration was inferred from the difference in newly bound AM receptors along the gradient. Computer simulations with the refined AM chemotaxis model, which is described in the Section 2 and depicted in Supplementary Figure S2, enabled us to narrow down the regime of relevant parameters in terms of the diffusion coefficient, the degradation rate and the secretion rate of the postulated chemokine.

#### 3.3.1. First-passage-times are mainly determined by diffusion coefficients and secretion rates

**Figure 4:**
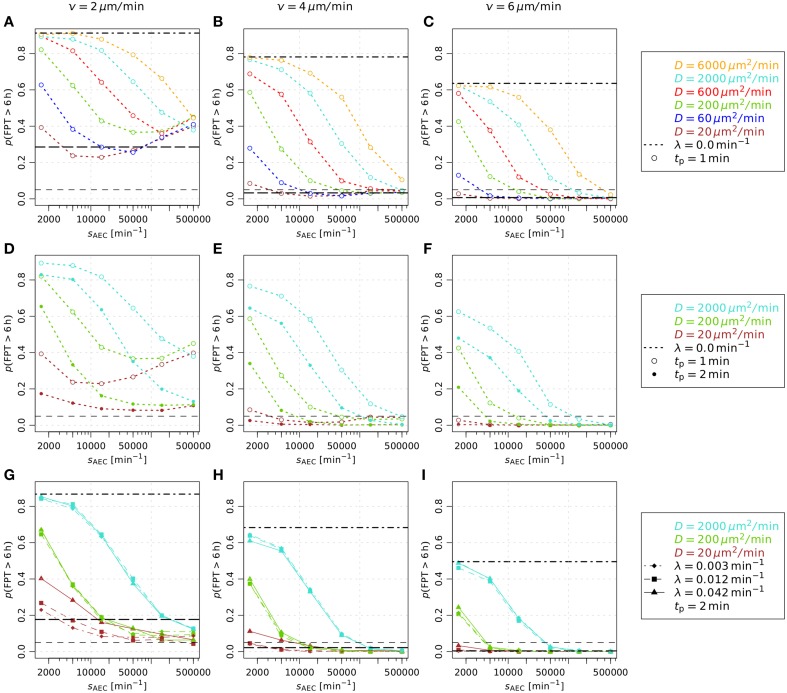
**Analysis of first-passage-time distributions varying the macrophage related parameters migration speed and persistence time and varying the chemokine related parameters diffusion coefficient, secretion rate and degradation rate**. In each subfigure **(A–I)** the fraction of first-passage-times above 6 h, *p*(FPT > 6 h), is plotted against the secretion rate of the AEC associated with the fungal conidi. The calculation of this fraction is based on the first-passage-time distribution which was derived performing 1000 first-passage-time simulations per parameter configuration. The results of the present study are compared to the biased persistent random walk (*long-dashed black line*) and the persistent random walk (*dashed-dotted black line*) by Pollmächer and Figge (2014). The *short-dashed black line* denotes the threshold *p*(FPT > 6 h) = 0.05. In **(A–C)** the focus is on the variation of diffusion coefficients, **(D–F)** show the results for different persistence times *t*_p_ and **(G–I)** demonstrate the influence of the degradation rate λ.

We measured first-passage-times in the alveolus, i.e., the time of first contact between AM and the conidium (see Video S3), in order to determine the requirements on the chemokine properties for a successful discovery of the fungal pathogen before the onset of germination (see Section 2.4.2 for details). First-passage-times were computed for 864 different parameter combinations (see Supplementary data in Supplementary Material) and for each combination 10^3^ simulations of the *A. fumigatus* infection scenario were performed to obtain statistically firm results. From the distributions of first-passage-times, we computed the fraction of first-passage-times above 6 h, *p*(FPT > 6 h), where 6 h were chosen based on the typical time period required for *A. fumigatus* germination. The results are presented in Figure 4 and demonstrate, in agreement with our previous study (Pollmächer and Figge, 2014), that AM with migration speed *v* = 2 μm/min exceeded the first-passage-time of 6 h in more than 5 % of the simulations for all parameter combinations (see short-dashed lines in Figures 4A,D,G). A comparison of Figures 4A,D shows that an increase in the persistence time from *t*_p_ = 1 min to *t*_p_ = 2 min was always associated with a decrease of *p*(FPT > 6 h). Next, we found that taking molecular degradation into account did not have a strong impact on *p*(FPT > 6 h), as can be observed by comparing Figures 4D,G for *t*_p_ = 2 min. These observations remain qualitatively the same for higher migration speeds of AM, see Figures 4B,E,H for *v* = 4 μm/min and Figures 4C,F,I for *v* = 6 μm/min. However, higher migration speeds of AM do have a quantitative impact on *p*(FPT > 6 h).

The dashed-dotted and long-dashed lines in Figure 4 indicate the values of *p*(FPT > 6 h) for AM performing, respectively, a persistent random walk and a biased persistent random walk, as previously simulated in Pollmächer and Figge (2014). The persistent random walk of AM always marks an upper limit for *p*(FPT > 6 h), i.e., first-passage-times are on average always decreased in the presence of chemotaxis, as could be expected for a low concentration of chemokines in the alveolus. On the other hand, compared to the biased persistent random walk model the performance of the chemotaxis model could yield lower values for *p*(FPT > 6 h), depending on the combination of parameters. In particular, we found that this is the case for combinations of a relatively high secretion rate and a relatively low diffusion constant. Note that the probabilistic rule for biased persistent random walk as previously simulated in Pollmächer and Figge (2014) was coupled to the direction of the shortest path from the AM to the AEC associated with the conidium. Occasionally, AM could leave the alveolus through a pore of Kohn if one of them was along the respective path of migration. In the present approach the frequency of this event was reduced, due to preferred AM migration in the direction of the chemokine gradient, which generally pointed away from pores of Kohn (see Videos S2 and S3). In summary, the diffusion coefficient and the secretion rate were again found to be the most important parameters, whereas the value of the degradation rate had only minor impact on *p*(FPT > 6 h) (see Figures 4G–I).

**Figure 5:**
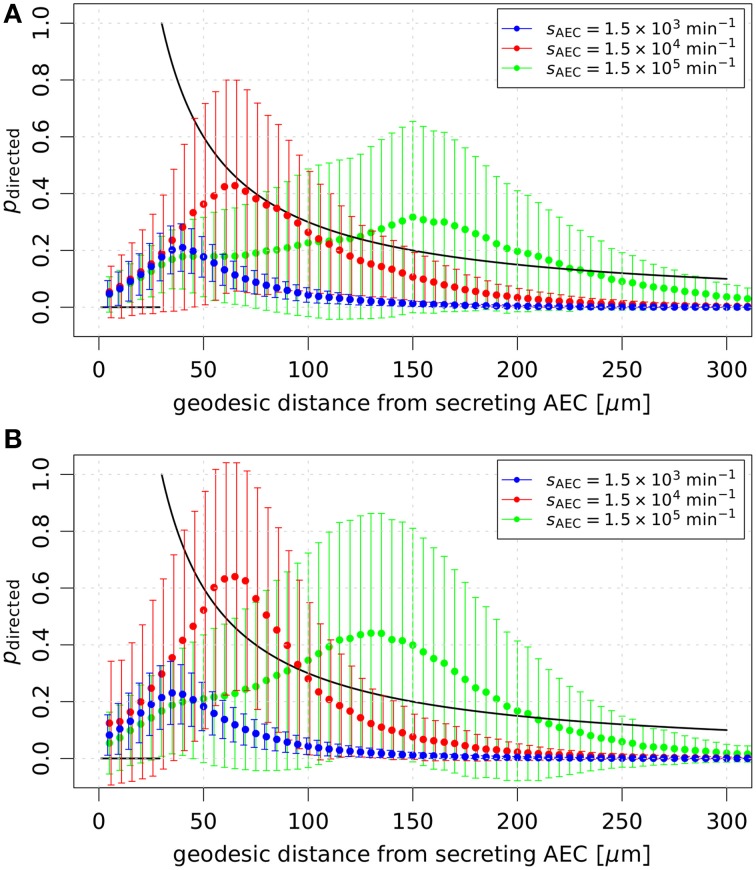
**Probabilities of directed AM migration over the geodesic distance from the AEC associated with the conidium**. The mean and standard deviation of the probability *p*_directed_ are shown in the absence of chemokine degradation for the diffusion coefficient *D* = 60 μm^2^/min with AM directional persistence time *t*_p_ = 1 min. In **(A)** AM migrate with speed *v* = 2 μm/min and in **(B)** with speed *v* = 4 μm/min. Averages and standard deviations were determined using the probabilities of directed AM migration that were drawn over the whole simulation time in all simulation runs. The present results are compared to the probabilistic rule for directed migration (*solid black line*) used in Pollmächer and Figge (2014).

Interestingly, we observed a minimum of *p*(FPT > 6 h) as a function of the secretion rate for various diffusion coefficients in the case of AM migration speed *v* = 2 μm/min and persistence time *t*_p_ = 1 min (see Figure 4A). This system behavior reflects the fact that an optimal concentration of chemokines exists for an efficient guidance of AM. The value of the optimal concentration is determined by the interplay of several factors, e.g., the secretion rate, diffusion coefficient and degradation rate of the chemokine as well as the number of AM receptors and their dynamics of binding, internalization and re-expression. For example, a too high chemokine concentration is associated with a low number of unbound AM receptors limiting the adaptation of AM migration along the chemokine gradient. We further analyzed this situation by computing the probability of directed AM migration for different secretion rates and for AM migration speeds *v* = 2 μm/min and *v* = 4 μm/min. The resulting probability distributions are shown in Figure 5 as a function of the geodesic distance of AM from the AEC associated with the conidium. We found that optimal values of *p*(FPT > 6 h) in Figure 4A correspond to probability distributions with a narrow and peaked maximum (see red curves in Figure 5). For a constant diffusion coefficient, lower secretion rates were associated with less prominent maxima in the probability distribution (see blue curves in Figure 5), which in turn increased *p*(FPT > 6 h). On the other hand, higher secretion rates were associated with extended and flat maxima at relatively large geodesic distances from the boundary of the secreting AEC (see green curves in Figure 5). It should be noted that the profiles of the determined probability distributions are the results of various factors, such as the chemokine concentration and the receptor dynamics of AM. For example, in the case of high secretion rates, many AM receptors were already bound to the chemokine at early time points due to its relatively high concentration in the alveolus. As a result, AM were guided to the AEC associated with the conidium relatively early in time. However, the relatively high concentration of chemokines also had the adverse effect that the number of free AM receptors was decreased at distances close to the secreting AEC. Consequently, fewer events of receptor-ligand binding lead to relatively low probabilities for directed AM migration and ultimately increased *p*(FPT > 6 h).

**Figure 6:**
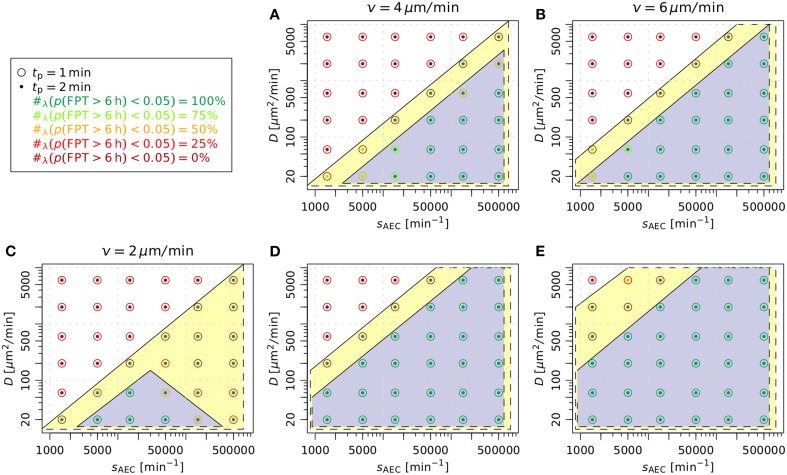
**Evaluation of parameters related to the AM chemoattractant based on the fraction of first-passage-times above 6 h**. **(A,B)** summarize the results from Figure 4, where AM insertion followed a uniform random distribution over the length of the boundaries of the three-quarter alveolus. The case *v* = 2 μm/min was left out as all parameter combinations lead to *p*(FPT > 6 h) ≥ 0.05. In **(C–E)** AM were inserted into the alveolus following a gradient-based probability distribution over the line elements belonging to the boundaries. The highlighted areas denote the regimes of parameters leading to timely detection of the conidium for the directional persistence times *t*_p_ = 1 min (*blue area*) and *t*_p_ = 2 min (*yellow area*) of AM under different migration speeds *v*. The variable #_λ_ (p(FPT > 6 h) < 0.05) denotes the fraction of simulated degradation rates that lead to *p*(FPT > 6 h) < 0.05 for a specific combination of parameters *D* and *s*_AEC_.

An overview of the relevant combinations of model parameters for successful detection of the *A. fumigatus* conidium by AM is given in Figure 6 for AM migration speed *v* = 4 μm/min (A) and *v* = 6 μm/min (B). As in (Pollmächer and Figge, 2014), we considered a parameter combination to be successful, if the value of *p*(FPT > 6 h) was below five percent. Interestingly, the ratio between the secretion rate and the diffusion coefficient, *s*_AEC_/D, was found to subdivide the parameter space into regimes of successful and unsuccessful parameter combinations. For *v* = 4 μm/min and *t*_p_ = 1 min, successful detection occurred for *s*_AEC_/D ≥ 250 μm^−2^ (see Figure 6A). Moreover, with increasing directional persistence time and/or migration speed of AM this threshold was found to be systematically reduced. While the combinations (*v*, *t*_p_) = (4 μm/min, 2 min) and (*v*, *t*_p_) = (6 μm/min, 1 min) both shared the condition *s*_AEC_/D ≥ 75 μm^−2^, for (*v*, *t*_p_) = (6 μm/min, 2 min) this threshold *s*_AEC_/D was lowered to the value 25 μm^−2^. To summarize, we found that the successful detection of the conidium by AM required the ratio between the secretion rate and the diffusion coefficient to be above a specific threshold, whereas the degradation rate had only minor impact on the first-passage-time (see Figures 4, 6).

#### 3.3.2. Gradient-based recruitment of AM increases relevant parameter regimes

Next, we studied a modification of AM insertion into the system at the boundaries, i.e., the alveolar entrance ring and the pores of Kohn. Previously, AM entered the three-quarter alveolus following a uniform random distribution over the length of the line elements belonging to all boundaries (Pollmächer and Figge, 2014). In the modified setup, we accounted for the time-evolution of the chemokine gradients at the boundaries by specifying probabilities for AM insertion according to the respective gradient strengths. In other words, AM insertion is more likely at boundaries with higher chemokine gradients (see Section 2.4.3 for details).

In Figures 6C–E it can be seen that gradient-based recruitment of AM generally increased the regime of parameter combinations for successful detection. At AM speeds of 4 μm/min and 6 μm/min the ratio of secretion rate to diffusion coefficient was systematically reduced (see Figures 6D,E). In contrast to the case where AM insertion was not gradient-based, in the present case a successful detection was also achieved at the migration speed of 2 μm/min for specific parameter combinations (see Figure 6C). Interestingly, for the AM parameters (*v*, *t*_p_) = (2 μm/min, 1 min) the subdivision of the parameter space into regimes of successful and unsuccessful parameter combinations was not only determined by the ratio *s*_AEC_/D. We checked that *p*(FPT > 6 h) has a dependence on the secretion rate similar to the simulations in the absence of gradient-based AM insertion (see Figure 4A). However, in the present case the minimum of *p*(FPT > 6 h) reached values below the five percent threshold for a limited range of the secretion rates that gave rise to the triangular region (see Figure 6C, blue area). The virtual infection model with gradient-based recruitment underlines the importance of chemokine-induced AM insertion points relative to the conidium position, as the results display a beneficial effect for the immune response of the host.

## 4. Discussion

In this study, we implemented a hybrid agent-based model (hABM) for *A. fumigatus* infection in human alveoli under physiological conditions to decipher the properties of a chemoattractant responsible for guiding alveolar macrophages (AM). The multi-scale simulations account for the dynamics at the cellular and molecular level, as well as the kinetics of binding, internalization and re-expression of chemokine receptors on AM. To scan the parameter space for combinations of parameters that ensure the timely detection of a conidium in the alveolus, we performed more than a million simulations of virtual infection scenarios in the experimentally relevant regimes. We were able to show that successful detection of the pathogen by AM is governed by the choice of five experimentally undetermined parameters: migration speed *v* and directional persistence time *t*_p_ of AM as well as the secretion rate *s*_AEC_, diffusion coefficient *D* and the degradation rate λ of the chemokine.

Simulations of the chemokine dynamics on the inner surface of the alveolus with its peculiar boundary conditions were performed using an efficient and accurate finite difference method on Voronoi cells (Sukumar, 2003) to solve the reaction-diffusion equation on an unstructured triangular Delaunay grid with close-to-equidistant grid points. We first studied the chemokine profile in steady state under varying conditions in an empty three-quarter spherical alveolus. Our results show that, depending on the diffusion coefficient of the chemokine, the time until a steady state is reached can vary from several minutes for *D* ≥ 2000 μm^2^/min to several hours for *D* ≤ 60 μm^2^/min. This revealed that our previous study, where the chemokine dynamics was simplified by a probabilistic rule, is limited to infection scenarios in the limit of high diffusion coefficients (Pollmächer and Figge, 2014). In contrast, using the present approach we are in the position to study *A. fumigatus* infection from the onset of chemokine secretion by alveolar epithelial cells (AEC) induced by the conidium and extending over the time period of establishing a concentration profile until the conidium is successfully found by one of the AM.

Since it was shown that AM require chemotactic cues in order to timely detect the conidium before the start of germination (Pollmächer and Figge, 2014), we here developed the hABM to account for the spatio-temporal concentration of chemokines in the alveolus. We implemented the receptor-kinetics chemotaxis model of Guo et al. (2008) for AM migration on a grid with high spatial resolution to capture the spherical alveolar surface with the pores of Kohn. The chemotaxis model accounts for the binding of G protein-coupled receptors on the surface of AM to the AEC-derived chemoattracting ligands in the alveolar lining layer (surfactant). In general, eukaryotic cells are able to sense spatial differences in receptor occupation along the chemokine gradient by their relatively large size of at least 10 μm (van Haastert and Postma, 2007). In order to sense shallow gradients in the chemokine concentration of 1–5 %, chemotactic cells are in addition able to sense temporal differences in receptor occupation, which increases the signal-to-noise ratio and implies higher probabilities of polarization directed along the gradient (van Haastert and Postma, 2007). We extended the chemotaxis model of Guo et al. (2008) by implementing AM sensing of the cumulated number of newly bound receptors over directional persistence times. This approach advances our previously applied phenomenological chemotaxis model, which was based on a constant function for the distance-dependent gradient strength (Pollmächer and Figge, 2014). In the present study, AM were enabled to sense dynamically changing local chemokine gradient strengths, which implicitly contained morphological information, e.g., concentration gradients pointing away from boundary elements. Simulations of the virtual infection scenario indicated that the present AM chemotaxis model unifies the random migration and chemotactic migration modes of our previous study in one model. In particular, we showed that persistent random walk was resembled for relatively low chemokine concentrations in the alveolus.

The computation of the first-passage-time, i.e., the duration until the conidium is detected by an AM for the first time, revealed the relative importance of the parameters associated with the chemokine distribution: the diffusion coefficient and the rate of chemokine secretion by the AEC associated with the conidium turned out to have a major impact, whereas chemokine degradation played a minor role. In particular, we found that the AEC secretion rate and the diffusion coefficient had counteractive effects regarding the average concentration of chemokines in the surfactant, i.e., decreasing the secretion rate lowered the average concentration whereas decreasing the diffusion coefficient increased it. Chemokines are diffusing in the alveolar lining layer (surfactant), which shields AM from the alveolar airspace, reduces surface tension and provides immunoregulatory proteins (Herzog et al., 2008; Hasenberg et al., 2013). In comparison with chemokine diffusion in water, the relatively high viscosity of the surfactant (Alonso et al., 2005) has the crucial effect to reduce the diffusivity of chemokines and by that to lower the AEC secretion rate required for the timely detection of the pathogen. We found the ratio of the AEC secretion rate to the diffusion coefficient, *s*_AEC_/D, to be the main indicator for the outcome of the infection scenario. For specific values of the AM migration speed and directional persistence time, this ratio subdivided the parameter space into regimes of successful and unsuccessful parameter combinations, whereas this separation was only weakly depending on relevant degradation rates. The degradation rate showed to have some impact in virtual infection scenarios with relatively low diffusion coefficients, which was also the case in the simulations associated with the steady state analysis. Thus, decisive reduction of the chemokine amount available to AM due to molecular degradation is only of importance for a highly viscous surfactant. The specific morphology of human alveoli plays an important role in this regard as chemokine reduction was also a consequence of chemokine absorption at the pores of Kohn and the alveolar entrance ring. A relative dominance of chemokine decrease due to alveolar boundaries was determined for relatively high diffusion coefficients, whereas relatively low diffusion coefficients were accompanied with relatively high chemokine degradation. This was attributed to reduced molecule motion for reduced diffusion coefficients, thus, on average molecules remained in the alveolus for a longer time period before leaving through the alveolar boundaries. As observed in our previous study (Pollmächer and Figge, 2014), AM required a minimal migration speed of at least 4 μm/min to discover the fungal conidium before the onset of germination. However, as shown in the present study, assuming a recruitment of AM from neighboring alveoli that was based on the local chemokine gradient, an average speed of 2 μm/min was as well successful for a specific subset of parameter combinations. This finding is particularly interesting, because the actual AM migration speed in the alveolus is not known today, but is typically expected to be low (Hasenberg et al., 2013). Generally, our results show that the communication between different types of host immune cells and their reaction to threatening invaders needs to be finely tuned in order to mount and orchestrate a fast and adequate response.

The specific chemokine and AM receptor that are involved in the directed migration are not known today. It is well-known that AM express, for example, the chemokine receptor CXCR2 (Miller et al., 2003) that binds to the cytokine IL-8. Moreover, the presence of complement proteins in the surfactant yields the cleavage product C5a, and this anaphylatoxin is a potential candidate for which AM chemoattraction was observed (Farrell et al., 1990; Zipfel and Skerka, 2009). Resting conidia of *A. fumigatus* activate the complement system entirely by the alternative pathway (Kozel et al., 1989). Upon activation, C3 is cleaved into C3b and C3a, with C3b opsonizing the fungal surface and increasing uptake rates by macrophages (van Lookeren Campagne et al., 2007). Furthermore, C3b induces cleavage of C5 which leads to the production of the prominent proinflammatory and chemoattracting cytokine C5a (Brakhage et al., 2010). However, it is also known that resting *A. fumigatus* conidia reduce the impact of the complement cascade by binding complement regulatory proteins—such as factor H, FHL-1, CFHR-1, C4BP and plasminogon—and by that reducing the deposition of C3b molecules on their surface (Behnsen and Hartmann, 2008). These data suggest that single conidia do both trigger and counteract the complement cascade, such that the mediated stimulus of chemoattraction and inflammation is relatively weak and spatially confined. Nevertheless, it is conceivable that these signals can be detected by the AEC associated with the conidium and that this cell responds with the secretion of the chemokines for AM recruitment. Supporting evidence for this hypothesis is provided by a study of rat AEC of type II: binding of C5a to these cells lead to increased expression of the C5a receptor on the AEC surface and to the production of macrophage inflammatory protein-2 as well as neutrophil-chemoattractant-1 (Riedemann et al., 2002).

Our computational approach to investigate *A. fumigatus* infection complements wet lab experiments. *In vivo* measurements suffer from the circumstance that they can only be carried out with high doses of conidia that do not reflect the physiological condition of daily inhalation rates of a few thousand conidia (O'Gorman and Fuller, 2008; Pollmächer and Figge, 2014). The agent-based modeling approach allows studying the early immune response, i.e., we modeled a setting with those immune cells that are resident in alveoli and performed virtual infection simulations to low numbers of conidia in a physiologically reasonable host-setting. Simulations enabled narrowing down the experimentally relevant regime of parameters to a subset of potential parameter combinations for healthy individuals. These predictions may initiate further wet lab investigations that should focus on quantitative aspects of the early immune response, e.g., the relative contributions of the complement system and the alveolar epithelial cells to the daily challenge with *A. fumigatus* or the identification of the specific chemokine for AM and the rate at which it is secreted by AEC. Furthermore, if possible by sophisticated imaging techniques in the future, it will be highly interesting to determine values of AM migration speed and migration mode in their natural environment to clarify their general role in the immune response, e.g., as compared to neutrophil migration in the alveolus (Mircescu et al., 2009).

In the context of studying fungal infections, image-based systems biology is able to serve as a well-founded framework with iterative cycles of exchange between experiment and theory and involves imaging, quantitative characterization and modeling of infection processes (Medyukhina et al., 2015). Methods for image-analysis of fungal-host interactions (Mech et al., 2011; Kraibooj et al., 2014; Brandes et al., 2015) and parameter-free classification of cell-tracks (Mokhtari et al., 2013) have been developed over the recent years and have paved the way for the quantification and extraction of the information contained in image- and video data. Furthermore, different individual-based modeling approaches were successfully carried out in combination with automated image-analysis to test hypotheses and to draw predictions that might be tested in future experimental research (Tokarski et al., 2012; Mech et al., 2013; Hünniger et al., 2014). Experimental studies including live-cell imaging in alveolar ducts would give the opportunity to refine, to review and to extend the present virtual infection model.

## Author contributions

Conception and design of the investigation and work: JP, MTF. Contribution of materials and computational resources: MTF. Data processing, implementation and application of the computational algorithm: JP. Evaluation and analysis of the results: JP, MTF. Drafting the manuscript and revising it critically for important intellectual content and final approval of the version to be published: JP, MTF. Agreement to be accountable for all aspects of the work in ensuring that questions related to the accuracy or integrity of any part of the work are appropriately investigated and resolved: JP, MTF.

### Conflict of interest statement

The authors declare that the research was conducted in the absence of any commercial or financial relationships that could be construed as a potential conflict of interest.
