# Supplementary material for: Deciphering chemokine properties by a hybrid agent-based model of Aspergillus fumigatus infection in human alveoli
Source: Front Microbiol. 2015 May 28;6:503. doi: 10.3389/fmicb.2015.00503 (PMC4446573; doi:10.3389/fmicb.2015.00503)
Supplement: Supplementary file 4 [file DataSheet1.PDF]

Supplementary Material: Deciphering chemokine properties by a hybrid agent-based model of *Aspergillus fumigatus* infection in human alveoli

Johannes Pollmächer [1,2] **and Marc Thilo Figge** [1,2,*]

[1] *Applied Systems Biology, Leibniz-Institute for Natural Product Research and Infection Biology – Hans Knöll Institute, Jena, Germany*
[2] *Friedrich Schiller University Jena, Jena, Germany*

Correspondence*:
Marc Thilo Figge
Applied Systems Biology, Leibniz-Institute for Natural Product Research and Infection Biology – Hans Knöll Institute, Adolf-Reichwein-Str. 23, Jena , 07749, Germay, thilo.figge@hki-jena.de

Computational Systems Biology of Pathogen-Host Interactions

1 SUPPLEMENTARY DATA

1.1 SIMULATED PARAMETER COMBINATIONS

Simulations of the virtual *A. fumigatus* infection scenario were carried out using 864 different parameter combinations of the chemokine diffusion coefficient D, the chemokine secretion rate s_{AEC}, the degradation rate λ, the AM migration speed v and the AM directional persistence time t_{p}. We performed simulations with 10^3 repetitions for each parameter configuration

$$(D, s_{\mathrm{AEC}}, \lambda, v, t_{\mathrm{p}}) \quad \in \quad \mathcal{D} \times \mathcal{S}_{\mathrm{AEC}} \times \Lambda \times \mathcal{V} \times \mathcal{T}_{\mathrm{p}} \quad ,$$

with the following sets of parameters for the distinct system variables:

$$\begin{aligned}
\mathcal{D} &= \{20, 60, 200, 600, 2000, 6000\}\, \mu\mathrm{m}^2/\mathrm{min} \quad , \\
\mathcal{S}_{\mathrm{AEC}} &= \{1\,500, 5\,000, 1.5 \times 10^4, 5 \times 10^4, 1.5 \times 10^5, 5 \times 10^5\}\, \mathrm{min}^{-1} \quad , \\
\Lambda &= \{0, 0.003, 0.012, 0.042\}\, \mathrm{min}^{-1} \quad , \\
\mathcal{T}_p &= \{1, 2\}\, \mathrm{min} \quad , \\
\mathcal{V} &= \{2, 4, 6\}\, \mu\mathrm{m}/\mathrm{min} \quad .
\end{aligned}$$

Parameters related to the chemokine were scanned logarithmically and AM migration parameters were scanned linearly in their respective experimental ranges.

2 SUPPLEMENTARY TABLES AND FIGURES

Figure S1. Macro- and microscopic view on the surface discretization of the three-quarter alveolus. **(A)** A grid with 5000 Voronoi cells, where grid points of category *boundary* and *outside* are left blank. **(B)** Neighbourhood-relationship between the grid points of the triangulated lattice with Voronoi cells providing the measures that are required to numerically solve the PDE.

Figure S2. Chemotaxis model of the mth alveolar macrophage (AM) in the computer simulations. AM (*light green*) are initialized with a population of R_0 free chemokine receptors at $t = 0$ or on boundary insertion into the alveolus during simulation time. Free receptors of each AM bind with the chemokine ligands at grid points covered by the corresponding shape in order to sense the chemokine gradient in the surfactant. Over the time segment of each directional persistence time AM register the accumulation of receptor-ligand bindings which is then used to determine the local AM gradient. Finally, the probability to migrate in the direction of the local gradient is proportional to the difference in bound receptors between the front and the rear of the cell. Thus, the velocity of AM not necessarily point in the direction of the chemokine gradient, but displays a bias depending on the strength of the gradient. See Materials and Methods section of the main document for further details.

3 SUPPLEMENTARY VIDEOS

Video S1. Dynamic overlay of the three-quarter alveolus simulation environment and the Delaunay triangulation of the alveolar surface using $10\,000$ grid points.

Video S2. Time evolution of chemokine distribution in a human three-quarter alveolus in the absence of alveolar macrophages. The alveolar epithelial cell (*yellow*) associated with the conidium of *A. fumigatus* (*red*) secretes chemokines at a rate of $1.5 \times 10^4\,\text{min}^{-1}$, which diffuses with $D = 200\,\mu\text{m}^2/\text{min}$ and $\lambda = 0\,\text{min}^{-1}$. Isolines (*white*) of the concentration values $\{2, 1, 0.5, 0.25\}\,\mu\text{m}^{-2}$ are plotted proportional to their respective values with different sizes. The video is sampled at a rate of 12 frames per second and the time between two frames corresponds to 0.5 minutes real time.

Video S3. Virtual infection scenario of *A. fumigatus* (*red*) in a human three-quarter alveolus. Alveolar macrophages (*green*) migrate with speed $v = 4\,\mu\text{m}/\text{min}$ and with directional persistence time $t_\text{p} = 2\,\text{min}$. The alveolar epithelial cell (*yellow*) associated with the conidium of *A. fumigatus* (*red*) secretes chemokines at a rate of $1.5 \times 10^4\,\text{min}^{-1}$, which diffuses with $D = 200\,\mu\text{m}^2/\text{min}$ in the absence of degradation. Isolines (*white*) of the concentrations $\{2, 1, 0.5, 0.25\}\,\mu\text{m}^{-2}$ are plotted proportional to their respective values with different sizes. The video is sampled at a rate of 12 frames per second and the time between two frames corresponds to 0.5 minutes real time.
