# Supplementary figures and images for: Deciphering chemokine properties by a hybrid agent-based model of Aspergillus fumigatus infection in human alveoli
Source: Front Microbiol. 2015 May 28;6:503. doi: 10.3389/fmicb.2015.00503 (PMC4446573; doi:10.3389/fmicb.2015.00503)

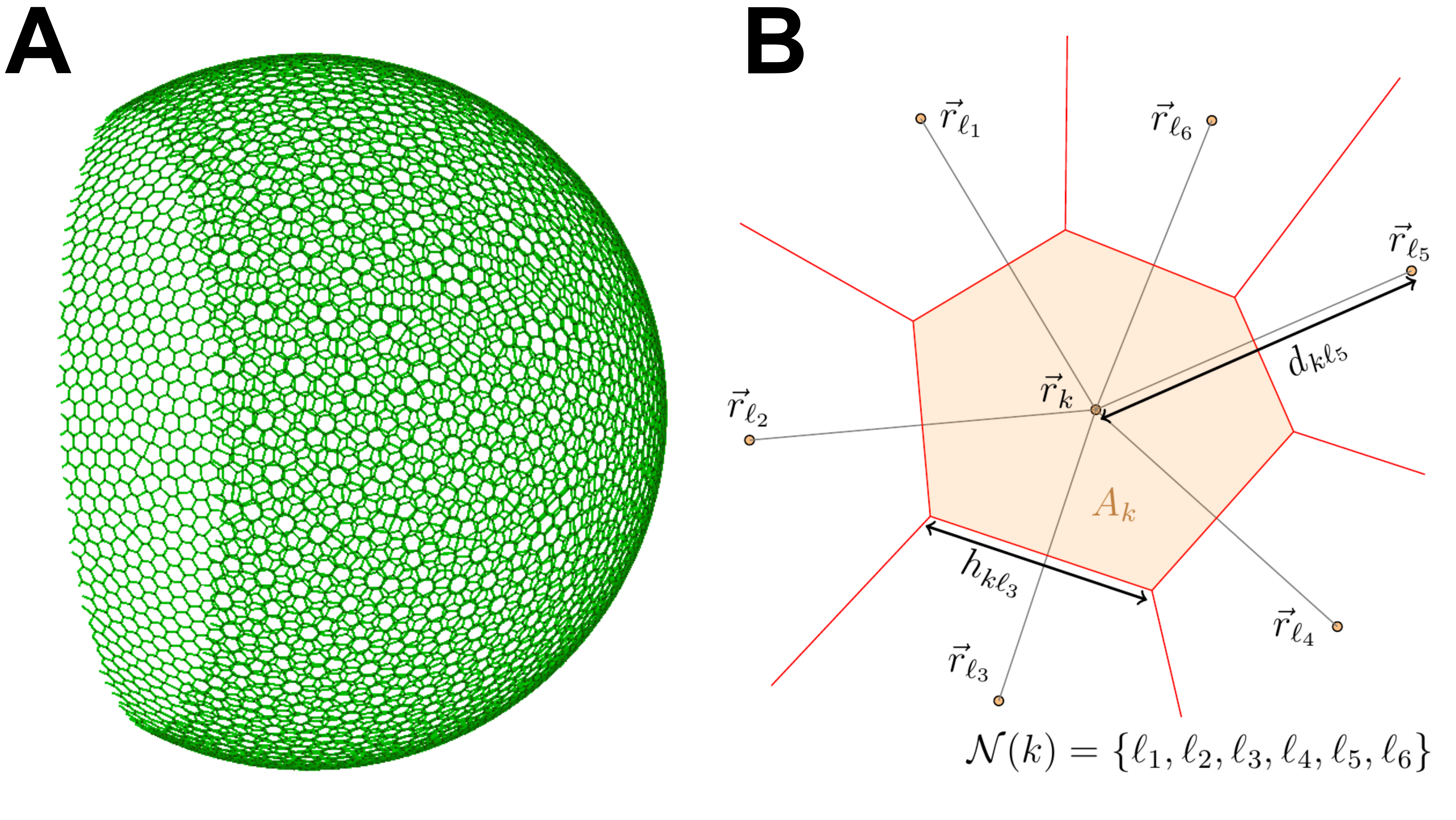

Supplement: Supplementary file 5 [file Image1.TIF]

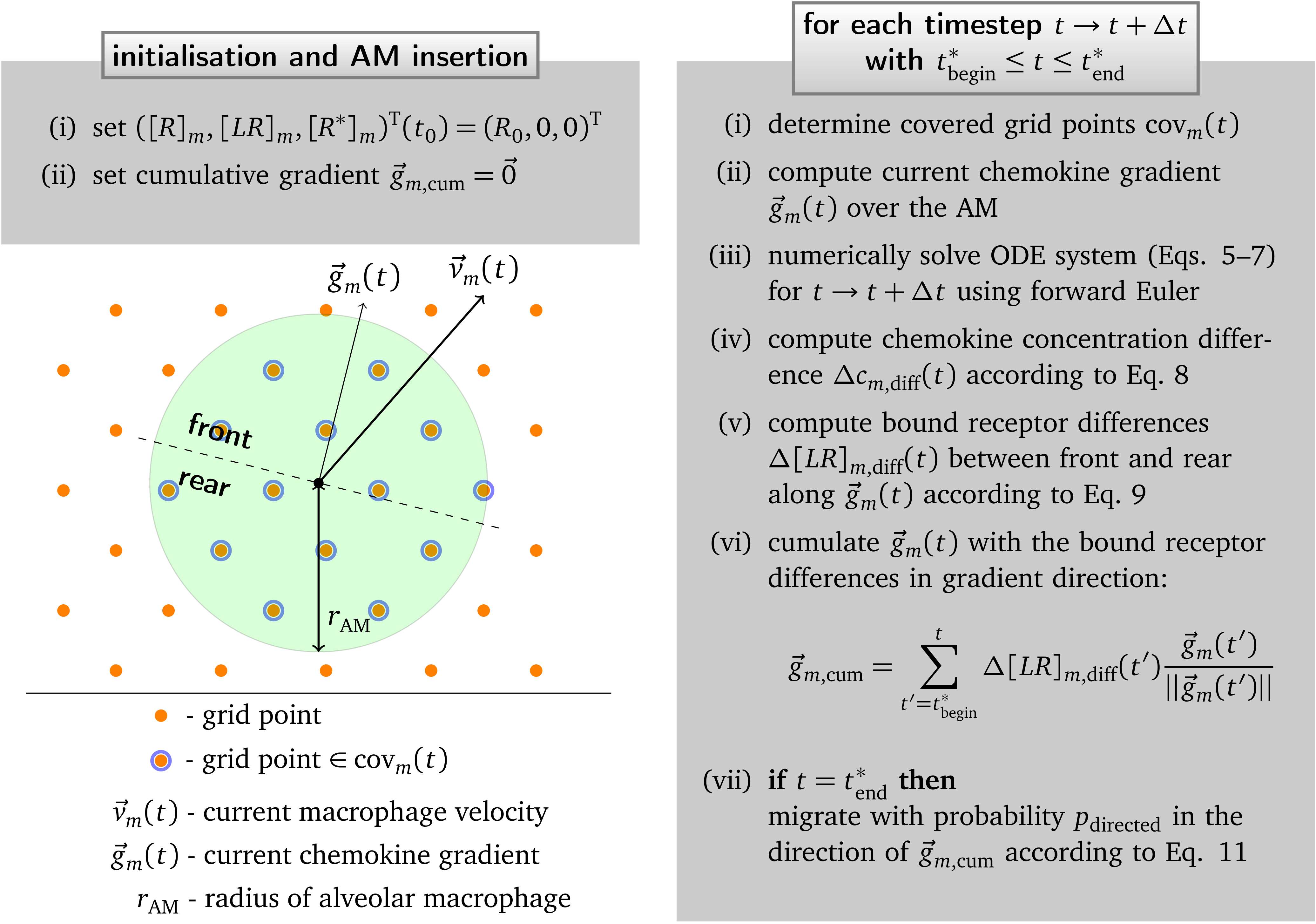

Supplement: Supplementary file 6 [file Image2.TIF]
